# Benefits of Stimulus Exposure: Developmental Learning Independent of Task Performance

**DOI:** 10.3389/fnins.2016.00263

**Published:** 2016-06-17

**Authors:** David B. Green, Jocelyn Ohlemacher, Merri J. Rosen

**Affiliations:** Department of Anatomy and Neurobiology, Northeast Ohio Medical UniversityRootstown, OH, USA

**Keywords:** learning, experience, auditory perception, gap detection, development, adolescent, adult, prepulse inhibition

## Abstract

Perceptual learning (training-induced performance improvement) can be elicited by task-irrelevant stimulus exposure in humans. In contrast, task-irrelevant stimulus exposure in animals typically disrupts perception in juveniles while causing little to no effect in adults. This may be due to the extent of exposure, which is brief in humans while chronic in animals. Here we assessed the effects of short bouts of passive stimulus exposure on learning during development in gerbils, compared with non-passive stimulus exposure (i.e., during testing). We used prepulse inhibition of the acoustic startle response, a method that can be applied at any age, to measure gap detection thresholds across four age groups, spanning development. First, we showed that both gap detection thresholds and gap detection learning across sessions displayed a long developmental trajectory, improving throughout the juvenile period. Additionally, we demonstrated larger within- and across-animal performance variability in younger animals. These results are generally consistent with results in humans, where there are extended developmental trajectories for both the perception of temporally-varying signals, and the effects of perceptual training, as well as increased variability and poorer performance consistency in children. We then chose an age (mid-juveniles) that displayed clear learning over sessions in order to assess effects of brief passive stimulus exposure on this learning. We compared learning in mid-juveniles exposed to either gap detection testing (gaps paired with startles) or equivalent gap exposure without testing (gaps alone) for three sessions. Learning was equivalent in both these groups and better than both naïve age-matched animals and controls receiving no gap exposure but only startle testing. Thus, short bouts of exposure to gaps independent of task performance is sufficient to induce learning at this age, and is as effective as gap detection testing.

## Introduction

Detecting rapid temporal changes in acoustic stimuli is essential for speech perception. In particular, certain phonemes are distinguished solely on the gap, or temporal interval, between consonant release and the onset of voicing (Steinschneider et al., 1994; Eggermont, 1995a,b). The ability to detect gaps in sound has an extended developmental trajectory in both humans and animals (Dean et al., 1990; Trehub et al., 1995; Friedman et al., 2004; Walker et al., 2006; Moore et al., 2011; Sanes and Woolley, 2011). Across the lifespan, gap detection thresholds (GDT) correlate with speech processing abilities (Snell and Frisina, 2000; Muluk et al., 2011). In prelinguistic children, GDTs are highly predictive of future language perception abilities, providing a valuable diagnostic tool and suggesting an early time window for intervention (Benasich et al., 2006; Muluk et al., 2011). As such, it is important to delineate the normal trajectory of gap detection with age and whether it can be improved by experience or training.

We examined both the developmental trajectory of gap detection abilities and the effect of experience with gaps using prepulse inhibition (gap-PPI) of the acoustic startle response (ASR). It is generally considered that learning in sensory perception requires attention or task engagement (Kral and Eggermont, 2007; Seitz and Dinse, 2007; Schreiner and Polley, 2014). PPI, a measure of detection rather than perception, is not influenced by attentional factors provided the interval between stimulus and startle is ≤ 60 ms (Li et al., 2009). Despite this, there is evidence that learning may occur during PPI (Ropp et al., 2014). Over repeated testing days, adult rats exhibited increasingly sensitive detection of 20 ms gaps (Crofton et al., 1990). In a developmental gap-PPI study, similar learning occurred in juvenile rats, although there was no control for improvement due to maturation of the auditory system (Friedman et al., 2004).

A more robust test of learning without attention is stimulus exposure in the absence of task performance. In the ASR testing paradigm, this equates to presentation of gaps without presentation of the startle to test detection. Animals are provided no explicit motivation to direct their attention to the gaps in this paradigm. In operant conditioning tests in adult and juvenile animals, non-attended stimulus exposure did not produce learning, even with a regimen matching that of active training (Sarro and Sanes, 2011; Vollmer and Beitel, 2011). Rather than improvement, chronic exposure to auditory stimuli during developmental critical periods can have long-lasting, detrimental effects on both non-attentive detection and attentive perception of auditory stimuli (Sun et al., 2011; Zhu et al., 2014). This exposure also induces abnormal sensory cortical representations (Zhang et al., 2002; Chang and Merzenich, 2003; Nakahara et al., 2004). Outside of these critical periods, chronic sound exposure typically has no effect (Zhang et al., 2002; Nakahara et al., 2004; Schreiner and Polley, 2014; but see Pienkowski and Eggermont, 2012; Zhou and Merzenich, 2012).

To date, the effects of short bouts of stimulus exposure in a non-attentive, sensory detection task have not been investigated. There are, however, reports of beneficial effects of stimulus exposure uncoupled with task performance. Developmental exposure to an enriched environment can both enhance normal perception and remediate perceptual performance deficits caused by noise rearing (Xu et al., 2009; Zhu et al., 2014). Perceptual learning can occur when task-irrelevant auditory or visual stimuli are presented during an attentive task (Seitz and Watanabe, 2003; Amitay et al., 2006), and additional stimulus exposure can enhance perceptual learning when presented within several minutes of an auditory training task (Wright et al., 2010).

To assess developmental learning without explicit attention, in this study we apply a gap-PPI paradigm that does not permit attentional modulation of detection (Li et al., 2009). This allows us to determine whether experience with gap-PPI can improve gap detection abilities during development, beyond improvement due to normal maturation. Furthermore, we identify the effect of passive stimulus exposure on gap detection learning by comparing normal gap-PPI testing against stimulus exposure without task performance, during development. Finally, we assess whether these non-attentional learning effects during development persist into adulthood.

## Materials and methods

### Subjects

**Figure 1:**
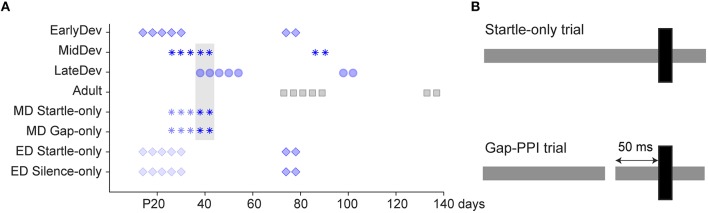
**Experimental design. (A)** Timeline of test sessions for each group, where darker symbols are gap-PPI sessions and lighter symbols are startle-only, gap-only, or silent control sessions. The *gray shaded* region refers to sessions compared in Figure 4. As the four ED groups were pooled into one for analysis, we represent here the group that began on P14. **(B)** Schematic of startle-only and gap-PPI stimuli. Gray bars depict background noise and black bars depict startle stimuli.

All procedures relating to the maintenance and use of animals were approved by the Institutional Animal Care and Use Committee at Northeast Ohio Medical University. Mongolian gerbils (*Meriones unguiculatus*) ranging from postnatal (P) day 12–137 underwent behavioral gap detection testing. Males (*n* = 44) and females (*n* = 51) from multiple litters were housed with littermates in a 12 h light/dark cycle. For the main experiments testing gap detection across development, four experimental groups spanned the developmental period from hearing onset to adulthood: Early Development (ED1, 2, 3, and 4), *n* = 14 per subgroup; Mid Development (MD), *n* = 11; Late Development (LD), *n* = 13; and Adult, *n* = 16. Aside from the experimental sound exposures, all animals were exposed only to typical ambient noises encountered in standard laboratory and animal facility environments; they were raised in separate cages within the same room in the animal facility. Testing for the four ED subgroups commenced at P12, 13, 14, and 16 to assess potential maturational differences over a period where sensory deprivation revealed discrete critical periods for synaptic and intrinsic properties of auditory cortical neurons (Mowery et al., 2014). There were no significant differences between any of these 4 subgroups, so data were pooled into one ED group, represented as starting at P14 in Figure 1A. Testing for the MD, LD and Adult groups began at P26, P38, and P73, respectively. An initial 5 sessions of gap-PPI testing were performed, with 3-day intervals (e.g., the MD group was tested at P26, 30, 34, 38, and 42). Subsequently, all groups were retested twice as adults, 60 and 64 days after initial testing onset. Weaning occurred at P30, so most sessions for ED animals and the first session for MD animals were conducted prior to weaning.

As the testing procedure involved multiple elements (testing environment, gap stimuli, startle stimuli, age of exposure), several control groups received treatments to separate the effects of these aspects of testing (Figure 1A). Two ED control groups were tested for 5 sessions beginning at P14, with intersession intervals identical to the experimental groups, but differing in stimulus presentation. ED Startle-Only (*n* = 14) received exposure to the testing environment with continuous background noise but without gaps, and startle stimuli identical to those presented to the experimental groups. ED Silence-Only (*n* = 16) received exposure to the testing environment without any auditory stimuli (no startle, background noise, or gaps). Both groups were subsequently tested using the standard gap-PPI protocol 60 and 64 days after testing onset. Two MD control groups received 3 sessions of exposure to the testing environment beginning at P26, differing in stimulus presentation. MD Gaps Only (*n* = 14) received the same gaps in noise presented to the experimental groups but no startle stimuli. MD Startle Only (*n* = 15) received continuous background noise without gaps but with startle stimuli identical to those presented to the experimental groups. Both groups were then tested for two sessions, with intersession intervals identical to the experimental groups, with the standard gap-PPI protocol.

### Behavioral testing

Gap detection abilities were assessed using PPI of the ASR, where some type of prepulse inhibits the startle response. The strength of inhibition corresponds with an animal's detection of the prepulse. Here, the prepulse was a silent gap in background noise (gap-PPI). The procedure has been described previously (Longenecker and Galazyuk, 2012). Briefly, animals were placed inside an acoustically transparent plastic restrainer, on an accelerometer plate in a sound attenuated, anechoic booth. Two separate speakers in each booth presented either background white noise at 60 dB SPL (presented from the front) or a startling stimulus at 110 dB SPL (presented from the top; Kinder Scientific Inc.). We presented 190 trials in pseudorandom order. Of these, 95 trials were startle–only (Figure 1B, top), with a startle stimulus of 20 ms broadband noise at 110 dB SPL, 1 ms rise/fall time. The remaining 95 were gap trials, where the startle stimulus was preceded by a silent gap in the noise background of either 2, 3, 5, 7, or 10 ms, with 19 trials of each gap duration. At the beginning of each session, 5 startle-only trials were presented (not included in analysis) to habituate the startle-only and PPI responses to a steady-state level (Ison et al., 1973). As shown in Figure 1B, bottom, the interstimulus interval, i.e., the time between gap termination and startle stimulus onset, remained a constant 50 ms as the duration of the gap changed. An interval of this duration was chosen to eliminate the involvement of any attentional factors (Li et al., 2009), allowing testing for learning exclusive of attention. Furthermore, the time period between trials (intertrial interval) was varied pseudorandomly between 12 and 18 s so that animals could not anticipate trial onset.

Testing was conducted once every 4 days. This intersession interval allowed us to (1) span the developmental period, (2) overlap age groups to control for the effects of age and experience independently, and (3) reduce habituation effects over sessions (Parisi et al., 1979). Habituation is the gradual reduction of the startle-only response magnitude, i.e., the ASR. The magnitude of the ASR depends on the amplitude of the startle stimulus, so it was necessary to choose an amplitude that would reveal a clear reduction of the ASR as a result of PPI. Preliminary experiments were conducted on separate groups of juvenile and adult gerbils to create startle-response functions across startle amplitude. From these functions, a startle amplitude of 110 dB SPL was chosen to provide the greatest potential dynamic range for all ages (Longenecker and Galazyuk, 2012).

### Data analysis

A gap detection threshold (GDT) was calculated for each animal at each session. First, the response magnitude to the startle stimulus was assessed using the RMS in the time window 20–50 ms after startle stimulus onset. For all trial types (startle-only or any gap duration), the distribution of RMS responses had a strong positive skew. A log_10_ transform was found to be the best at generating a normal distribution of RMS responses within each trial type, as assessed using the Anderson-Darling test. Then using a bootstrap method, for each trial type we determined the RMS response threshold at which a reduction in startle was considered statistically significant. To do so, the median values of the peak transformed responses for startle only and each gap duration were plotted, and a cubic spline was fitted to this plot, creating a detection function. To find where that function crossed a detection criterion, the transformed startle-only values were sampled with replacement 10,000 times to generate a normal distribution, from which 95% confidence intervals were calculated. The lower confidence interval was the value where a reduction in startle indicated significant detection (Fechter et al., 1988). GDT was the level at which the fitted detection function crossed the lower confidence interval. Note that this analysis accounts for any inherent variability in startle magnitude within each animal.

In some instances, animals were unable to detect any of the experimental gaps, which is unsurprising as the longest gap presented was quite short (10 ms). This occurred most frequently in young animals and those with few sessions of experience. With development or experience, this occurred less often, suggesting that thresholds had improved to within the tested range. Pilot data indicate that animals of all ages tested can detect gaps >50 ms. Thus a lack of detection likely indicates a threshold higher than 10 ms. Conservatively, GDTs of 11 ms were substituted for sessions where gaps were not detected, for all analyses. Those GDTs are represented as NT (no threshold) in the figures. In Supplemental text, we repeat the analyses excluding these values. The results are generally consistent with the results with substituted GDTs presented in the main text.

GDTs are presented as both best performance over sessions and average performance over sessions. Best GDTs represent how well animals are capable of performing at a given age and level of experience, while average GDTs include variability, inherent both to early developmental stages and to the methodology. For most comparisons, the effects were robust to both best and average measurements.

## Results

### Developmental trajectory of gap sensitivity

**Figure 2:**
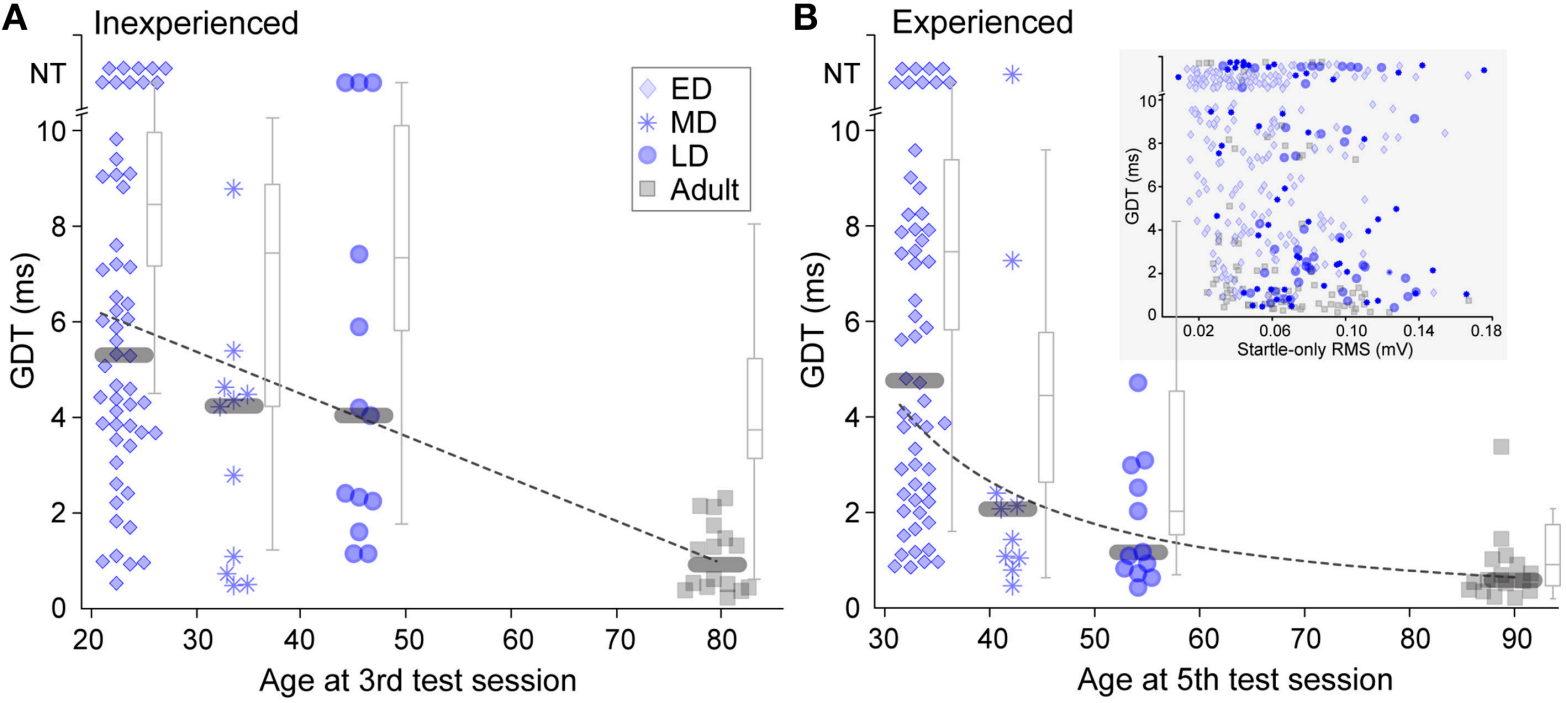
**Gap detection development with and without gap-PPI experience**. **(A)** Symbols depict best GDT and gray boxplots depict average GDT across sessions 1 through 3, providing a representation of inexperienced thresholds. NT = no threshold, indicating that the longest gap presented (10 ms) was not detected. **(B)** Symbols depict best GDT and gray boxplots depict average GDT across sessions 4 and 5, providing experienced thresholds. The improved thresholds in MD and LD groups show improvement based on experience. *Gray bars* depict median values. (Inset) Scatterplot for all age groups of the relationship between startle-only magnitudes and gap detection thresholds across age groups. There was no significant correlation. For boxplots, central mark is the median, edges are 25th and 75th percentiles, and whiskers extend to the most extreme data points excluding outliers.

GDTs for animals in each of the four age groups are shown in Figure 2. The data show gradual maturation of both thresholds and of across-animal variability (e.g., within a wide range, some young individuals performed at adult levels). Thresholds from animals inexperienced with gap-PPI are depicted in Figure 2A as the best GDT (blue symbols) or the average GDT (gray boxplots) across the first 3 sessions. This reveals a slow time-course of maturation for gap detection abilities, best fit by a linear trajectory (Pearson's correlation: best GDT: *r* = −0.48, *p* < 0.0001; average GDT: *r* = −0.54, *p* < 0.0001). GDTs were higher for all three developmental groups than for Adults (Kruskal–Wallis, best GDT: ED $\chi_{\left(1,\;71\right)}^{2}=$ 29.14, *p* < 0.0001; MD $\chi_{\left(1,\;26\right)}^{2}=$ 6.33, *p* < 0.02; LD $\chi_{\left(1,\;28\right)}^{2}=$ 13.26 *p* < 0.001; average GDT: ED $\chi_{\left(1,\;71\right)}^{2}=$ 25.6, *p* < 0.0001; MD $\chi_{\left(1,\;26\right)}^{2}=$ 5.4, *p* < 0.02; LD $\chi_{\left(1,\;28\right)}^{2}=$ 9.7, *p* < 0.002). In Figure 2B, experienced performance is depicted as the best GDT (blue symbols) or the average GDT (gray boxplots) across sessions 4 and 5, reflecting learning based on gap-PPI exposure. The Late-Dev (LD) group improved with experience (Kruskal–Wallis best GDT: $\chi_{\left(24\right)}^{2}=5.45,p=0.0195$; average GDT: $\chi_{\left(24\right)}^{2}=$ 11.3, *p* < 0.001), and the Mid-Dev (MD) group median detection shifted slightly, creating a function across development that was best fit by a logarithmic trajectory (Pearson's correlation: best GDT: *r* = −0.67, *p* < 0.0001; average GDT: *r* = −0.74, *p* < 0.0001). The Adult averages but not best performance improved with experience (Kruskal–Wallis best GDT: $\chi_{\left(30\right)}^{2}=$ 1.1, *p* = 0.3; average GDT: $\chi_{\left(30\right)}^{2}=$ 8.6, *p* < 0.005). This can be explained by the fact that the best thresholds in the first three sessions were already near ceiling. The Early-Dev (ED) group did not improve with experience within the range of gap durations tested (2–10 ms), but may have shown improvement if tested with longer gaps (Friedman et al., 2004). GDTs across animals were also much more variable during development than during adulthood, both with and without gap-PPI experience (Inexperienced, Interquartile GDT (ms): best ED 5.4; MD 3.8; LD 6.2; Adult 1.2; average ED 2.8; MD 4.6; LD 4.3; Adult 2.1. Experienced, Interquartile GDT (ms): ED 5.8; MD 2.2; LD 2.2; Adult 0.6; average ED 3.6; MD 3.1; LD 3.0; Adult 1.3). One component of this variability is that a subset of individuals at all ages performed at adult levels.

It is possible that determining gap detection using ASR, i.e., measuring the reduction in the magnitude of the startle response when a gap is present, might be confounded by the small mass of younger animals. Specifically, a smaller response magnitude to the startle-only stimulus produced by a lower mass reduces the dynamic range available for measuring a response reduction to the gap. To ensure that this was not confounding our results, we confirmed that the poorer thresholds elicited by younger animals were not an artifact of the measurement. There was no correlation between the magnitude of the startle-only response and gap detection thresholds within sessions (Figure 2B inset; Spearman's Rho using data from the first 5 sessions: ED: *r* = −0.1670, *p* = 0.009; MD: *r* = −0.2856, *p* = 0.035; LD: *r* = −0.0534, *p* = 0.728; Adult: *r* = −0.4050, *p* = 0.0002); in fact, values overlapped in all age groups.

### Developmental trajectory of gap detection learning

**Figure 3:**
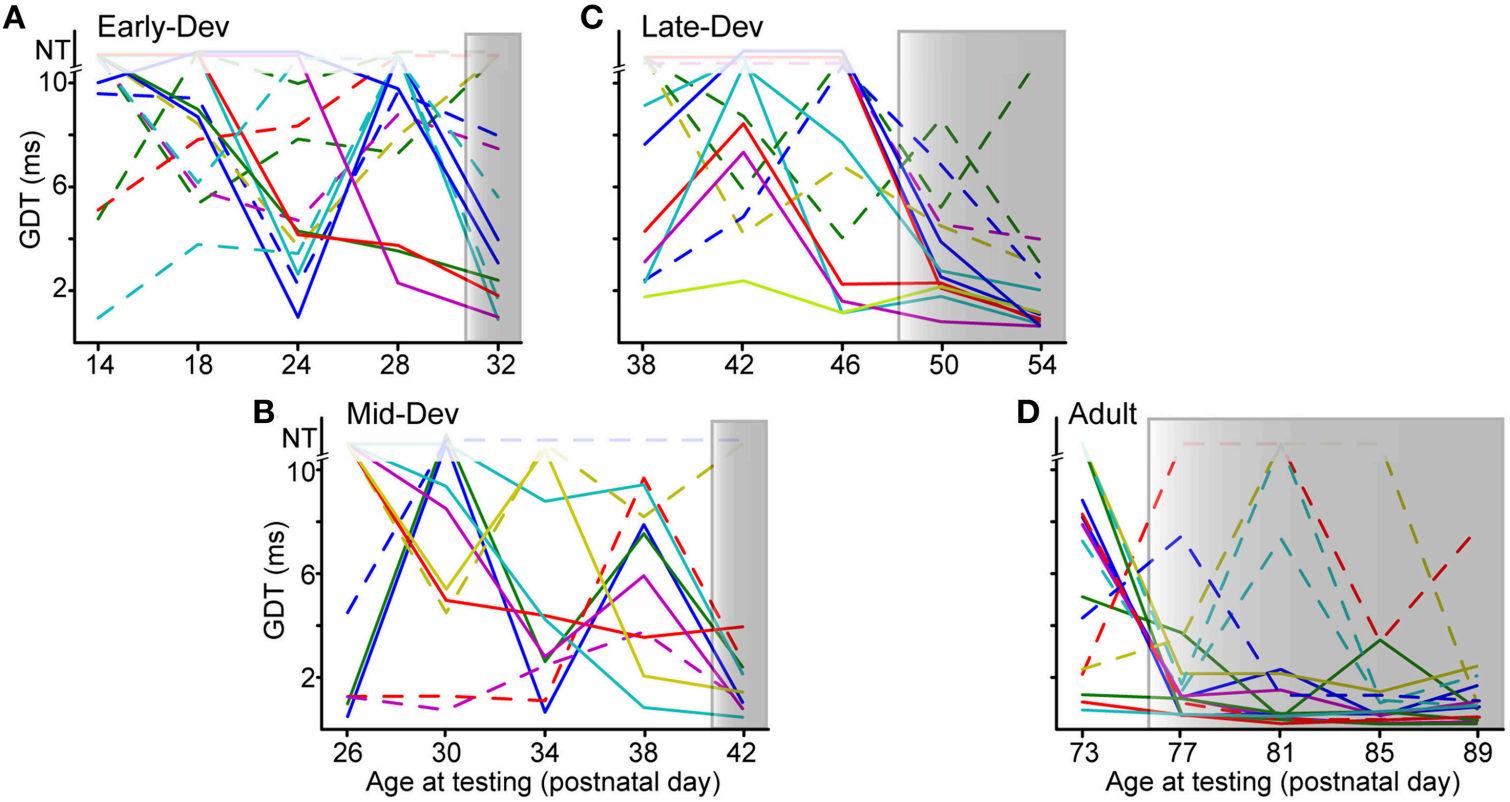
**The ability to improve with gap-PPI experience has its own developmental trajectory**. **(A–D)** Panels for each age group show individual trajectories of GDTs across sessions, arranged to depict sessions with overlapping ages across groups. *Shaded regions* are those sessions with significantly better GDTs compared with session 1. *Solid lines* are those animals showing trajectories of improvement, while *dashed lines* are those animals with more variable GDTs across sessions. The data displayed for ED is for a subset of the animals in that group (ED4) but the analysis was performed on data from all ED animals. NT = no threshold, indicating that the longest gap presented (10 ms) was not detected.

**Figure 4:**
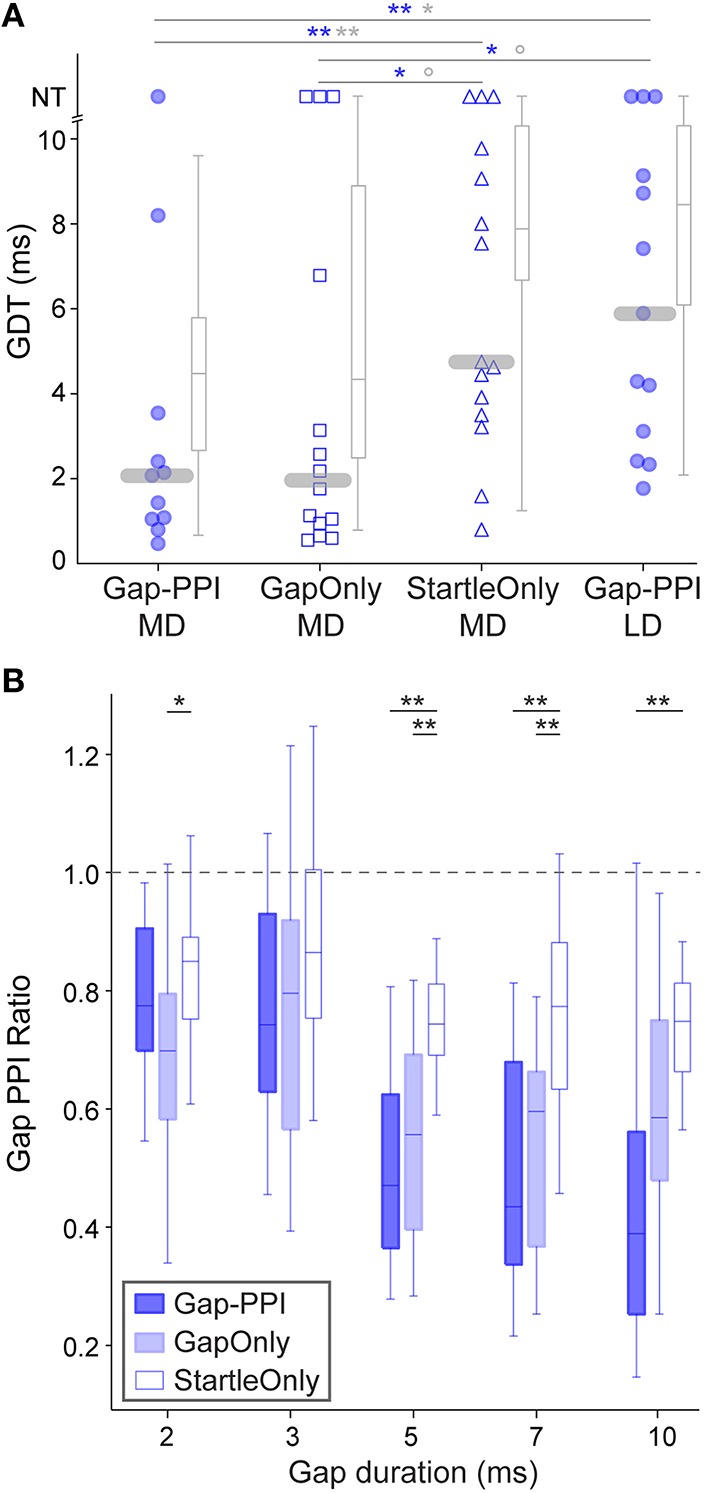
**Responses of age-matched animals that experienced various exposure regimens. (A)** Symbols depict best GDTs and gray boxplots depict average GDTs from two testing sessions in individual age-matched (P38-42) animals; age-matched sessions are those which are gray-shaded in Figure 1B. Gap-PPI LD animals were the naïve age-matched controls for the other groups which received different exposure regimens for three sessions prior to testing (gap-PPI, gap-only, or startle-only). Gap-PPI exposure and Gap-Only exposure were equivalently effective in reducing GDTs. Startle-Only exposure did not reduce GDTs, and their GDTs were equivalent to inexperienced Gap-PPI LD controls. NT = no threshold, indicating that the longest gap presented (10 ms) was not detected. Blue and gray significance symbols refer to best and average GDTs, respectively. ^**^p < 0.02; ^**^p < 0.05; °*p* < 0.1 **(B)** Response ratios across gap durations, normalized to startle-only responses (dotted line). The Gap-PPI and Gap-Only groups did not differ at any gap duration, while the Startle-Only group differed from either of these groups at most gap durations. ^**^p < 0.007; ^*^p < 0.03.

The improvement effect shown in Figure 2 is clarified by tracking individual performance within each age group. Figure 3 shows the performances of individuals throughout the initial 5 testing sessions, separated by age group. As shown, some animals improve (solid lines) while others have more variable GDTs across sessions (dashed lines). A linear mixed model ANOVA was used to assess improvement across sessions at a group level. We define improvement as lower thresholds. The shaded regions show the timepoint within each group at which thresholds were significantly lower than session 1, remaining lower for all subsequent sessions. Note that for clarity of presentation, panel A shows only the ED group that started on P16, though the analysis was performed on the pooled ED groups. ED and MD groups improved by the 5th session, the LD group by the 4th session, and the Adult group by the 2nd session. ED 5th session (*F*_(223)_ = 2.8, *p* = 0.001). MD 5th session (*F*_(40)_ = 3.35, *p* = 0.038). LD 4th session (*F*_(48)_ = 3.7 *p* < 0.002); 5th session (*F*_(48)_ = 5.0, *p* < 0.0001). Adult 2nd session (*F*_(60)_ = 3.7, *p* < 0.001); 3rd session (*F*_(60)_ = 2.9, *p* < 0.007); 4th session (*F*_(60)_ = 4.5, *p* = 0.001); 5th session (*F*_(60)_ = 4.8, *p* < 0.0001). There is still considerable within-animal variability across sessions, especially for the younger groups, suggesting a need for more experience to achieve consistent performance at younger ages. At a group level, this indicates a developmental trajectory for learning with non-attentional gap-PPI exposure. We confirmed that this improvement reflects learning rather than maturation of the auditory system by comparing age-matched animals with and without gap-PPI experience. The last two sessions of the MD group overlapped with the first two sessions of the LD group. Comparing performance from those sessions, the experienced MD animals had significantly better GDTs than the inexperienced LD animals (Figure 4A, Gap-PPI MD vs. Gap-PPI LD: Kruskal–Wallis, best GDT: $\chi_{\left(1,\;22\right)}^{2}=$ 6.54, *p* < 0.02; average GDT: $\chi_{\left(1,\;22\right)}^{2}\;=$ 4.87, *p* < 0.027). Thus, the improvement over testing sessions is due to gap-PPI experience rather than an effect of normal maturation.

### Effects of gap exposure alone on detection

Improvement over sessions indicates learning based on exposure to gap-PPI testing, but does not distinguish what aspect of testing induces learning. We tested two additional groups of animals to determine whether the ASR testing method or simply exposure to gaps in background noise (unpaired with the startle stimulus) was sufficient to induce learning. To examine learning across sessions, we chose animals age-matched to the MD group, which required several sessions of experience to achieve consistently improved performance. Startle-Only MD animals were exposed to ASR testing in background noise without gaps for 3 sessions, then gap-PPI tested for the final 2 sessions. Thresholds for Startle-Only MD animals were no better than age-matched LD animals, who were equivalently naïve in terms of gap exposure (best GDT: $\chi_{\left(1,\;26\right)}^{2}=$ 0.01, *p* = 0.93; average GDT: $\chi_{\left(1,\;26\right)}^{2}=$ 0.01, *p* = 0.93). This indicates that experiencing ASR testing alone did not improve GDTs. Gap-Only MD animals were exposed to background noise *with* gaps but *without* ASR testing for three sessions, then gap-PPI tested for the final two sessions. Based on the best of the two sessions, these Gap-Only MD animals were significantly better than both the age-matched Startle-Only and Gap-PPI LD groups (Kruskal–Wallis: Gap-Only MD vs. Startle-Only MD $\chi_{\left(1,\;27\right)}^{2}=$ 3.98, *p* < 0.05; Gap-Only MD vs. Gap-PPI LD $\chi_{\left(1,\;25\right)}^{2}=$ 4.1, *p* < 0.05). Averaging GDTs across those two sessions revealed marginally significant differences (Kruskal–Wallis: Gap-Only MD vs. Startle-Only MD $\chi_{\left(1,\;27\right)}^{2}=$ 3.15, *p* < 0.07; Gap-Only MD vs. Gap-PPI LD $\chi_{\left(1,\;25\right)}^{2}=$ 2.21, *p* < 0.1). Thus exposure only to gaps without PPI testing improved thresholds that animals were able to obtain. Moreover, Gap-Only animals did not differ from the Gap-PPI MD group, showing that exposure only to gaps produced GDTs equivalent to gap-PPI testing (Kruskal–Wallis: best GDTs: Gap-Only MD vs. Gap-PPI MD $\chi_{\left(1,\;23\right)}^{2}=$ 0.04, *p* = 0.85; average GDTs: Gap-Only MD vs. Gap-PPI MD $\chi_{\left(1,\;23\right)}^{2}=$ 0.09, *p* = 0.76). This effect is visible as well in a less processed representation of the data, across the range of gap durations we tested (Figure 4B): the magnitude of the startle reduction by the gap was equivalent between Gap-PPI and Gap-Only MD animals at all gap durations. In contrast, the control Startle-Only MD animals had less of a startle reduction than either of these groups at most gap durations. Thus exposure to gaps in background noise unpaired with startles is sufficient to induce gap detection learning, and is as effective as gap-PPI testing.

### Effects of early exposure on adult detection

**Figure 5:**
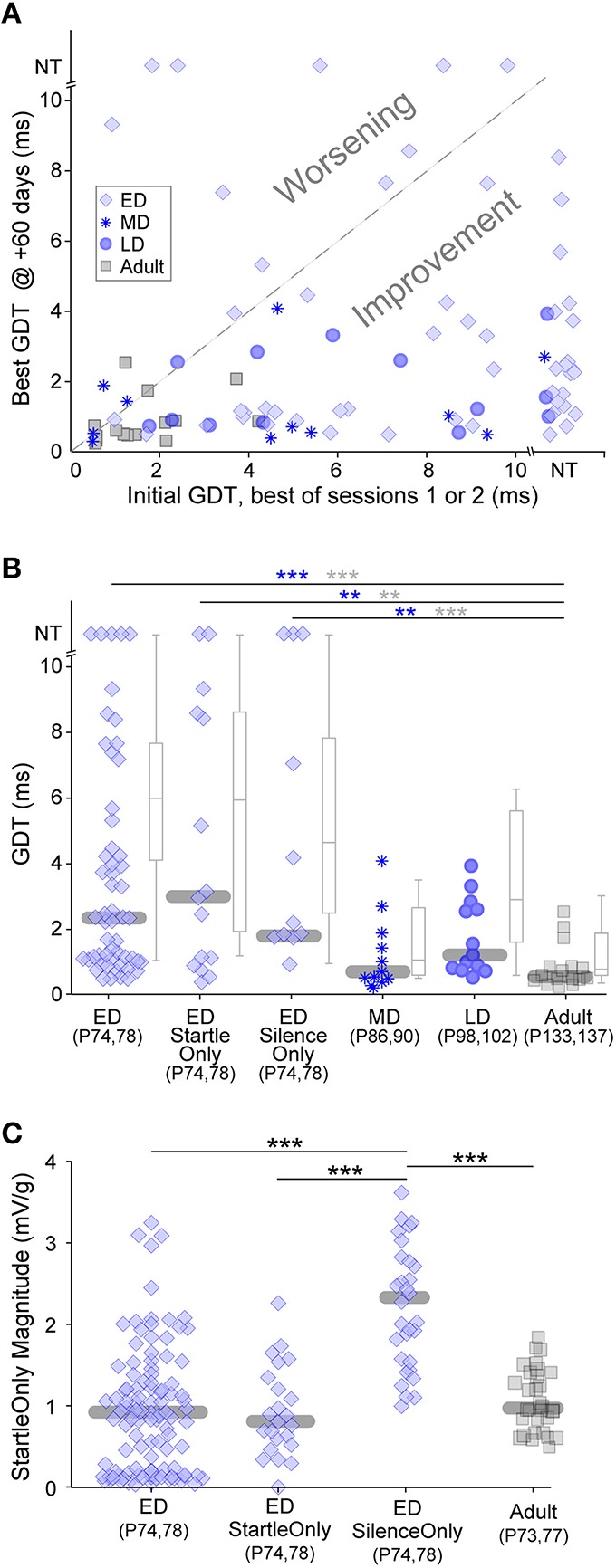
**Long-lasting effects of the age of experience**. All animals were tested 60 days after initial exposure. **(A)** Comparisons of initial and +60-day best GDTs separates animals that improved versus worsened based on early experience. Only ED animals worsened. NT = no threshold, indicating that the longest gap presented (10 ms) was not detected. **(B)** Symbols depict best GDTs and gray boxplots depict average GDTs across two testing sessions at +60-days, across developmental groups and two ED control groups. GDTs in all ED groups were higher than the Adult group. Blue and gray significance symbols refer to best and average GDTs, respectively. **(C)** The response magnitudes to startle stimuli without gaps were larger in the ED Silence-Only group than the other three groups serving as controls for the early testing experience. ^***^p < 0.004, ^***^p < 0.0002.

Chronic exposure to auditory stimuli can have long-lasting perceptual effects during development but not in adulthood (Schreiner and Polley, 2014). These are typically detrimental to perception. Both our gap-PPI and gap-only results indicate that in short bouts, non-attentive exposure causes improvement, raising the question of long term effects. We tested whether gap-PPI testing has differential long-lasting effects based on the developmental period of exposure, with the idea that early exposure could improve later adult gap detection more than adult exposure (Sarro and Sanes, 2011). ED, MD, LD and Adult groups were re-tested for two sessions, 60 days after the initial five sessions (see timeline in Figure 1B). Figure 5A compares each animal's initial GDT (the best of the first two sessions) with their final GDT (the best of the two +60-day sessions). This depicts adult gap detection (+60-day testing) after exposures at different developmental timepoints. The vast majority of animals improved (points below the diagonal) in the MD, LD and Adult age groups (Kruskal-Wallis, median improvement: MD: 4.1 ms, $\chi_{\left(1,\;20\right)}^{2}=$ 5.4, *p* < 0.02; LD: 3.5 ms, $\chi_{\left(1,\;24\right)}^{2}=$ 12, *p* < 0.001; Adult: 0.7 ms, $\chi_{\left(1,\;30\right)}^{2}=$ 7.4, *p* < 0.007). The final best GDTs of these three groups did not differ from one another, likely because all groups improved to ceiling performance (Figure 5B), obscuring any potential benefit of the age of initial exposure. Improvement for the majority of MD, LD, and adult animals holds true with the added variability included within averaged GDTs (Kruskal–Wallis, median improvement: MD: 6.8 ms, $\chi_{\left(1,\;20\right)}^{2}=$ 9.7, *p* < 0.002; LD: 5.6 ms, $\chi_{\left(1,\;24\right)}^{2}=$ 11.7, *p* < 0.001; Adult: 3.9 ms, $\chi_{\left(1,\;30\right)}^{2}=$ 9.8, *p* < 0.002).

In contrast to the other three groups, 20% of the ED animals worsened in adult retesting (Figure 5A, points above the diagonal), and the median ED GDTs during adult retesting were significantly higher than those of the Adult group (Figure 5B; ED vs. Adult: best GDT: $\chi_{\left(1,\;70\right)}^{2}=$ 19.3, *p* < 0.0002; average GDT: $\chi_{\left(1,\;70\right)}^{2}=$ 25.9, *p* < 0.0001). One possibility is that early exposure to the background noise with gaps may have caused a later gap detection deficit. To test this possibility, we added a control group (see timeline in Figure 1B). ED Startle-Only animals were exposed to five sessions of startle-only exposure during continuous background noise with no gaps, then gap-PPI tested at +60-days. At testing, their GDTs were equivalent to the ED group (ED vs. ED Startle-Only, best GDT: $\chi_{\left(1,\;68\right)}^{2}=$ 0.16, *p* = 0.69; average GDT: $\chi_{\left(1,\;68\right)}^{2}=$ 0.26, *p* = 0.61), and significantly higher than those of Adult-exposed animals (Figure 5B; ED Startle-Only vs. Adult best GDT: $\chi_{\left(1,\;30\right)}^{2}=$ 10.8, *p* < 0.004; average GDT: $\chi_{\left(1,\;30\right)}^{2}=$ 13.8, *p* < 0.002). Gap exposure thus did not cause worsening, as the Startle-Only animals worsened although they were not exposed to gaps.

Another possible explanation is that the startle exposure during testing may have induced hearing damage in the ED animals, as they were exposed to 110 dB SPL startle sounds as early as P12. To test this possibility, we added another control group (see timeline in Figure 1B). ED Silence-Only animals were simply placed in the startle enclosure without exposure to any auditory stimuli for five sessions, then tested at +60-days. At testing, their GDTs were equivalent to the ED group (ED vs. ED Silence-Only best GDT: $\chi_{\left(1,\;69\right)}^{2}=$ 0.01, *p* = 0.91; average GDT: $\chi_{\left(1,\;69\right)}^{2}=$ 1.05, *p* = 0.31), and significantly higher than those of Adult-exposed animals (Figure 5B; ED Silence-Only vs. Adult best GDT: $\chi_{\left(1,\;31\right)}^{2}=$ 14.0, *p* < 0.004; average GDT: $\chi_{\left(1,\;31\right)}^{2}=$ 13.1, *p* < 0.0003). These Silence-Only animals worsened although they were exposed to neither startle sounds nor gaps during development (their thresholds were only tested in adulthood). Thus, even if the other groups had hearing loss, this control indicates that worsening occurred in the absence of hearing loss. It is noteworthy that in all ED groups, many individuals performed as well as Adult exposed animals; our manipulations impaired later GDTs only in a subset of individuals.

An alternative explanation for the worsening involves non-auditory elements of testing. Maternal separation at a very young age has been demonstrated to induce long-lasting behavioral deficits and is a well-established stress model (Francis et al., 2002; Schäble et al., 2007; Nishi et al., 2014). One known effect of early acute stress is a higher response magnitude to startle-only stimuli (Bakshi et al., 1998). To test whether the ED animals displayed increased startle-only magnitudes, we compared the response magnitudes to startle-only stimuli across age-matched groups with different early experience (Figure 5C and timeline in Figure 1B). The age-match is necessary because animal mass affects startle magnitude, although it does not affect GDT as seen in Figure 2B inset. All ED groups experienced early testing and the possible stress it could induce. ED Silence-Only animals tested at +60-days, who were naïve to startle stimuli unlike the other two ED groups, had significantly higher startle response magnitudes than those two groups (ED Silence-Only vs. ED $\chi_{\left(1,\;70\right)}^{2}=$ 37.3, *p* < 0.0001; ED Silence-Only vs. ED Startle-Only $\chi_{\left(1,\;30\right)}^{2}=$ 27.8, *p* < 0.0001). This reflects a sensitized but transient response to startle, enhanced upon first experiencing startle sounds and back to normal levels after multiple startle sessions. Importantly, this large response magnitude was not due to naïvete to startles: the ED Silence-Only group had significantly higher startle response magnitudes than the age- and experience-matched Adult group who were equivalently naïve to startles ($\chi_{\left(1,\;29\right)}^{2}=$ 31.2, *p* < 0.0001).

## Discussion

Gap detection thresholds improved with maturation, although a subset of developing animals performed at adult levels (Figure 2). Both these observations agree with previous studies (Dean et al., 1990; Trehub et al., 1995; Fitch et al., 2008). Learning occurred across sessions, improving GDTs beyond those of age-matched controls. Based on this learning, we demonstrate for the first time a developmental trajectory for learning in animals, as younger animals required more sessions to achieve consistent improvement (Figure 3). This is consistent with human studies showing a developmental trajectory for training effects across age (Huyck and Wright, 2011). Most importantly, exposure to the gap stimuli without concurrent task performance produced learning equal to that induced by gap-PPI testing (Figure 4). Thus, an association between gap and startle is not required for this learning. Finally, in pre-weaned gerbils we demonstrated a detrimental effect of placement in the testing apparatus on gap detection that persisted into adulthood (Figure 5).

### Developmental trajectory of gap detection

Our data show that gap detection abilities are not mature until adolescence, consistent with previous reports in rodents (Dean et al., 1990; Friedman et al., 2004). In humans, gap detection maturation can extend into late childhood or early adolescence depending on stimulus parameter details (Davis and McCroskey, 1980; Irwin et al., 1985; Fischer and Hartnegg, 2004; Buss et al., 2014). Temporal processing in general has a long developmental trajectory, possibly due to an extended maturation of cortical circuitry. For example, in gerbil auditory cortex (ACx), responses to modulated signals are immature in P30-40 juveniles, correlating with behavioral detection (Rosen et al., 2010). ACx is required for detecting gaps < 50 ms (Ison et al., 1991; Threlkeld et al., 2008), likely via the response to gap termination: manipulating the balance of excitation and inhibition in the post-gap time window alters behavioral gap detection thresholds (Weible et al., 2014). Immature cortical inhibition is a candidate substrate (Chang and Merzenich, 2003) as interneuron numbers and VGAT clustering around GABAergic terminals are late to reach adult levels, following time-courses similar to that of behavioral gap detection (Ouellet and de Villers-Sidani, 2014; Hackett et al., 2015). In awake juveniles, cortical response latencies can be >30 ms longer than in adults (Rosen et al., 2010), such that the post-gap sound onset response that contributes to detection may not reliably occur within the interval between gap termination and startle.

Along with higher median GDTs, across-animal variability was greater in the younger groups. This is consistent with increased threshold variability in young animals (Sarro and Sanes, 2011) and children (Mednick et al., 2002; Garadat and Litovsky, 2007; Huyck and Wright, 2011; Moore et al., 2011) that has been demonstrated for many auditory tasks. Furthermore, within animals, session-to-session performance consistency was poor in our younger groups (Figure 3). This seems to be a feature of development, as performance consistency for temporal auditory processing tasks is poorer in younger animals and humans (Sarro and Sanes, 2010; Moore et al., 2011; Huyck and Wright, 2013). Higher variability can have several sources. Sensory representations may be immature and/or unreliable in younger animals, which has been demonstrated in ACx for temporally-varying stimuli (e.g., Eggermont, 1991; Rosen et al., 2010). Individuals within age groups may be at different maturational stages. This is supported by adult-like performance in a subset of individuals, both in children (Werner et al., 1992) and as we see here. Immature cognitive elements may also contribute, although this should be reduced by our gap-PPI task that excludes explicit attentional contributions.

### Sensory exposure is sufficient for gap detection learning

While many have shown that attention is essential for perceptual learning, and that sensory stimulation alone has no effect (Seitz and Dinse, 2007), evidence is accumulating for learning induced by stimulus exposure in the absence of reinforcement or attention. Our experiments used an interval between stimulus and startle at which, in human studies, signal detection is not enhanced by directed attention (Li et al., 2009). Additionally, a variable intertrial interval precluded anticipation of the gap, and the task had no trained elements. Thus, our gap-PPI should be uninfluenced by explicit attention to the gaps. Yet we and others have demonstrated that thresholds improve with PPI experience in both juveniles (Dean et al., 1990; Friedman et al., 2004) and adults (Crofton et al., 1990; Reijmers and Peeters, 1994). We have further demonstrated that stimulus exposure alone, without association with the startle, improves GDT as much as gap-PPI experience. This improvement is related to exposure to gaps: animals that experienced ASR in the presence of background noise without gaps did not learn. We interpret this gap exposure as passive. However, the animals may be in a heightened state of arousal while in the testing enclosure. Alternatively, they could be generally attending to the background noise with gaps, although they are provided with no motivation to do so. Either heightened arousal or non-specific attention may be important for the learning seen here.

These improved thresholds with gap-only experience indicate that some type of learning has occurred. We have shown here that pairing of the gap and startle is not necessary for eliciting improvement, indicating that associative learning is not involved. This contrasts with Crofton et al. (1990), who showed improvement only from sessions with paired gap and startle, but not with gap alone. Several differences in experimental design may contribute to this disparity. First, their protocol used an interval between gap and startle (170 ms) where PPI may be susceptible to attentional modulation (Elden and Flaten, 2002), which can contribute to associative learning (Pauli and O'Reilly, 2008). Second, they presented stimuli daily, while our sessions were separated by 3 days. Finally, they tested adults, excluding any potential developmental contribution. The learning we see might be age-dependent, as perception and ACx tuning in younger animals can be altered by extended passive stimulus exposure (de Villers-Sidani et al., 2007; Barkat et al., 2011), while adults require active engagement (Diamond and Weinberger, 1986; Bao et al., 2004; Blake et al., 2006) or neuromodulatory manipulation (Froemke et al., 2007).

While our results are not consistent with associative learning, they are similar to perceptual learning, where improvement occurs with stimulus exposure during training or performance. Active training can improve perceptual abilities via focused attention on particular stimulus features (Seitz and Dinse, 2007). Yet during perception tasks, presentation of irrelevant or non-attended stimuli enhances learning of those stimuli (Watanabe et al., 2001; Amitay et al., 2006; Wright et al., 2010), supporting the idea that task performance creates a sensitized state in which sensory stimulation can induce learning (Seitz and Dinse, 2007; Wright and Zhang, 2009). While there is no explicit attentional component to our gap-only exposure, animals may be generally attentive to the sound. Stimulus exposure during a state of arousal, such as that potentially elicited by placement in the startle enclosure, might induce a sensitized state, allowing gap-only exposure to induce learning. For example, unattended stimulus exposure during an unrelated task improved later auditory discrimination (Amitay et al., 2006), implicating general task-evoked arousal. Similarly, non-explicit stimulus elements, such as statistical structure in language or tone sequences, can be learned during unattended exposure where the context of the experiment is a potential arousal cue (Saffran et al., 1999).

For gap-PPI learning to occur, one or more neural regions involved in gap detection must interact with the circuitry underlying PPI, and learning-related plasticity must be able to affect the PPI circuitry. ACx is a strong candidate for involvement. First, cortical activity reflects auditory learning: tasks in which perceptual learning is demonstrated induce shifts in ACx tuning directly related to the relevant stimuli (Polley et al., 2006). Such alteration of tuning endures for hours, suggesting a long-lasting memory trace (Fritz et al., 2003). Second, ACx is required for gap-PPI, but not for PPI in general (Ison et al., 1991). Finally, ACx projects to three regions involved in PPI: inferior colliculus, superior colliculus, and pedunculopontine tegmental nucleus (Herbert et al., 1991; Li et al., 2009; Bajo et al., 2010; Schofield, 2010). There are thus multiple loci where learning-related plasticity in ACx could alter gap-PPI. Indeed, inactivation of excitatory neurons in A1 eliminates a previously induced PPI enhancement (Du et al., 2011). Despite this, the circuitry underlying gap-PPI differs in part from circuits involved in operant behavioral measures. In animals, operant measures are typically used to assess perceptual learning, raising the question of the relationship between such learning and that shown here. The gap detection thresholds produced by operant vs. gap-PPI measures in gerbils are quite similar (Wagner et al., 2003), yet other percepts (i.e., intensity discrimination) do not produce comparable thresholds: more sensitive performance was elicited by operant measures (Behrens and Klump, 2015). This discrepancy may be due to insufficient “training” via repeated PPI testing for this task. In rats, more complex tasks such as FM discrimination require more “training” (i.e., experience) with PPI than simple tasks such as gap detection (Fitch et al., 2008). Even here, adults improve after their first session. Future experiments are needed to elucidate whether plasticity induced by learning from repeated exposure to PPI resembles that induced by perceptual learning in operant tasks.

The plasticity induced by short periods of gap-only exposure may improve auditory processing deficits, particularly in young, prelinguistic children. Gap-detection deficits induced by cortical microgyria or chronic noise can be remediated by exposure to gap-PPI or chronic tones (Threlkeld et al., 2009; Jiang et al., 2015). As GDTs in children are predictive of later speech processing abilities, early remediation via simple stimulus exposure would be an easily implemented therapeutic option.

### Long-term effects of early gap-PPI testing

Developmental exposure to gaps might improve adult detection abilities. Juvenile gerbils trained on an operant conditioning task performed better as adults than age- and experience-matched controls (Sarro and Sanes, 2011). While our gap-PPI animals did not exhibit improved thresholds when retested in adulthood, this was likely due to a ceiling effect: even naïve adults by session two were reliably detecting the shortest gaps. Surprisingly, the ED group performed worse as adults compared with age-matched controls (Figure 5A). As several elements may underlie this deficit, we added two control groups to disambiguate them. The Startle-Only group was a control for potential gap-induced worsening. Perceptual worsening has been shown in subsets of children during training on auditory discrimination tasks (Huyck and Wright, 2011, 2013) and in adults trained on a texture discrimination task (Mednick et al., 2005). This worsening has been attributed to decreases in the stimulus-driven representation due to over-stimulation (Mednick and Drummond, 2008), raising the possibility that early exposure to gaps could induce poor performance as adults. However, even when presented with background noise excluding gaps, ED Startle-Only animals worsened (Figure 5B). This indicates that exposure to gaps was not necessary for the worsening. These animals did experience low-level background noise.

The Silence-Only group was a control for both exposure to background noise and for potentially damaging exposure to the startle sound. All the original groups received identical auditory experiences yet worsening occurred only in the ED animals. One explanation is that the ED animals may have been susceptible to hearing damage induced by exposure to the 110 dB SPL startles, while the older animals who did not worsen were resistant. This could be attributable to a potential immaturity of either lower or higher-level circuitry controlling the acoustic middle ear reflex (MER). The MER can protect against damage from loud sounds (Mukerji et al., 2010), although based on its ~25 ms latency in rats to ~100 dB SPL sounds (Murata et al., 1986), the MER would not be expected to provide protection for our 20 ms startle sounds. However, there is evidence that the muscles controlling the MER can be modified during ASR testing: the MER varies with level of arousal (Baust et al., 1964); the muscles involved receive modulatory inputs from a diversity of regions (Thompson et al., 1998; Lee et al., 2008); and ACx ablation drastically reduces the MER in cats (Baust et al., 1964), consistent with conscious control of the muscles by some humans (Mukerji et al., 2010). Furthermore, the middle ear muscles are active even to continuous sounds at levels lower than our background noise (Simmons, 1962; Murata et al., 1986), and non-auditory-evoked contractions occur during attentive states (Baust et al., 1964). Thus, activity of the middle ear muscles may be altered by modulatory inputs based on behavioral state, such as that experienced by animals confined in the testing apparatus. Auditory cortical regions are still immature in the ED group (Fitzgerald and Sanes, 2001), raising the possibility of reduced MER activation during ASR testing and potential hearing damage.

Surprisingly, worsening occurred in the ED Silence-Only group, despite being exposed only to silence and not tested with startles (Figure 5B). While we cannot exclude the possibility that the other animals may have had hearing loss, the presence of worsening in this group is a strong indicator that any potential hearing loss in the other groups is not the sole source of worsening, as the Silence-Only group experienced no potentially damaging sounds during testing and yet still showed worsening. Furthermore, they were raised in the same room and ambient auditory environment as all the other groups. The only difference in treatment between the ED Silence-Only and Adult groups was experiencing 5 h-long sessions confined in the testing apparatus early in development. The impaired adult performance must have arisen from some aspect of this experience. Our testing regimen involves restraint, handling, and maternal separation. All developmental groups were restrained and handled during testing, yet retested MD and LD groups did not worsen. While restraint is a stressor, brief (~1 h) maternal separation is also an established stressor for pre-weaned juvenile rodents (Francis et al., 2002; Schäble et al., 2007). Worsening in the ED groups may relate to the maternal separation experienced during testing.

When the three ED groups were retested as adults, ED Silence-Only displayed larger startle-only (i.e., ASR) magnitudes compared with age-matched Adult controls (Figure 5C). Increased ASR magnitudes occur as a result of acute stress in humans caused by the threat of electric shock (Grillon and Davis, 1997), or chronic stress in rats from isolation rearing (Bakshi et al., 1998; Li et al., 2008). The increased ASR magnitude reduces with repeated ASR testing (Bakshi et al., 1998). We see a similar effect here: the ED and ED Startle-Only groups had “normal” ASR magnitudes equivalent to age-matched Adult controls during retesting. These groups had already experienced 5 sessions of ASR testing during development, which would allow their ASR magnitude to return to normal levels by adult retesting. In contrast, both the Adult and ED Silence-Only groups experienced ASR for the first time as adults. ASR magnitudes increased only in the ED Silence-Only group, consistent with an effect of stress.

Stress early in development may have affected cortical circuitry, which could underlie the adult gap detection deficits. Stress has been shown to alter thresholds and task performance in auditory and somatosensory systems (Kadner et al., 2006; Pérez et al., 2013; Toya et al., 2014). Temporary separation and confinement of P14-26 rats (similar to ED Silence-Only) causes layer-specific dendritic shortening in ACx: shorter basal dendrites in 2/3 and shorter apical dendrites in layers 5 and 6 (Bose et al., 2010). Such changes could induce gap detection deficits via an altered network of excitatory neurons, as gap detection is known to rely on an interaction of inhibition and excitation in auditory cortex (Weible et al., 2014). We interpret the retested gap-PPI deficits as deficits in gap detection, although it cannot be ruled out that other aspects of the PPI circuitry may be involved (Larrauri and Schmajuk, 2006). If stress is indeed the source of the long-lasting effects we see here, our findings suggest that juvenile stress has more than one effect on PPI performance: higher GDTs and increased startle, with the former more persistent than the latter. We suggest that experiments involving even brief maternal separation may induce unintended stress effects that could confound the data.

## Author contributions

MR conceived the research; MR, DG, and JO designed the experiments; DG and JO collected the data; MR and DG analyzed the data; MR and DG wrote the paper.

### Conflict of interest statement

The authors declare that the research was conducted in the absence of any commercial or financial relationships that could be construed as a potential conflict of interest.
