# Supplementary material for: Benefits of Stimulus Exposure: Developmental Learning Independent of Task Performance
Source: Front Neurosci. 2016 Jun 17;10:263. doi: 10.3389/fnins.2016.00263 (PMC4911416; doi:10.3389/fnins.2016.00263)
Supplement: Supplementary file 1 [file DataSheet1.docx]

**Supplementary Results:

*All analyses exclude GDTs > 10ms.*** In the instances where excluding GDTs > 10ms changed the significance of the comparisons, those comparisons are shown in bold.

***Developmental trajectory of gap sensitivity***

GDTs for animals in each of the four age groups are shown in Figure 2. The data show gradual maturation of both thresholds and of across-animal variability (e.g., within a wide range, some young individuals performed at adult levels). Thresholds from animals inexperienced with gap-PPI are depicted in Figure 2A as the best GDT (blue symbols) or the average GDT (gray boxplots) across the first 3 sessions. This reveals a slow time-course of maturation for gap detection abilities, best fit by a linear trajectory (Pearson’s correlation: best GDT: r = ‑0.54, p < 0.0001; average GDT: r = -0.55, p < 0.0001). GDTs were higher for all three developmental groups than for Adults (Kruskal Wallis, best GDT: ED χ^2^(1,71) = 24.5, p < 0.0001; MD χ^2^(1,26) = 6.3, p < 0.02; LD χ^2^(1,28) = 9.7 p < 0.002; average GDT: ED χ^2^(1,71) = 32.1, p < 0.0001; MD χ^2^(1,26) = 9.4, p < 0.003; LD χ^2^(1,28) = 6.9, p < 0.009). In Figure 2B, experienced performance is depicted as the best GDT (blue symbols) or the average GDT (gray boxplots) across sessions 4 and 5, reflecting learning based on gap-PPI exposure. Based on average GDTs, **the Early-Dev (ED),** Late-Dev (LD) and **Adult** groups improved with experience, and the Mid-Dev (MD) group detection shifted down slightly (Kruskal Wallis: ED: **χ^2^(24) = 4.7, p < 0.04**, MD χ^2^(19) = 2.4, p = 0.1, LD χ^2^(24) = 8.1, p < 0.005, Adult **χ^2^(30) = 8.6, p < 0.005**), creating a function across development that was best fit by a logarithmic trajectory (Pearson’s correlation: r = -0.73, p < 0.0001). GDTs across animals were also much more variable during development than during adulthood, both with and without gap-PPI experience (Inexperienced, Interquartile GDT (ms): best ED 3.3; MD 3.8; LD 2.6; Adult 1.2; average ED 2.7; MD 4.6; LD 2.9; Adult 2.1. Experienced, Interquartile GDT (ms): ED 5.2; MD 1.4; LD 2.2; Adult 0.6; average ED 3.6; MD 3.3; LD 3.0; Adult 1.3). One component of this variability is that a subset of individuals at all ages performed at adult levels.

It is possible that determining gap detection using ASR, i.e. measuring the reduction in the magnitude of the startle response when a gap is present, might be confounded by the small mass of younger animals. Specifically, a smaller response magnitude to the startle-only stimulus produced by a lower mass reduces the dynamic range available for measuring a response reduction to the gap. To ensure that this was not confounding our results, we confirmed that the poorer thresholds elicited by younger animals were not an artifact of the measurement. There was no correlation between the magnitude of the startle-only response and gap detection thresholds within sessions (Figure 2B inset; Spearman’s Rho using data from the first 5 sessions: ED: r = ‑0.1670, p = 0.009; MD: r = -0.2856, p = 0.035; LD: r = ‑0.0534, p = 0.728; Adult: r = ‑0.4050, p = 0.0002); in fact, values overlapped in all age groups.

***Developmental trajectory of gap detection learning***

The improvement effect shown in Figure 2 is clarified by tracking individual performance within each age group. Figure 3 shows the performances of individuals throughout the initial 5 testing sessions, separated by age group. As shown, some animals improve (solid lines) while others have more variable GDTs across sessions (dashed lines). A linear mixed model ANOVA was used to assess improvement across sessions at a group level. We define improvement as lower thresholds. The shaded regions are based on analyses including GDTs > 10 ms, and show the timepoint within each group at which thresholds were significantly lower than session 1, remaining lower for all subsequent sessions. Note that for clarity of presentation, panel A shows only the ED group that started on P16, though the analysis was performed on the pooled ED groups. When excluding GDTs > 10 ms, ED and **LD** groups improved by the 5^th^ session, and the Adult group by the 2^nd^ session (ED 5^th^ session F(223) = 2.6, p = 0.01. **LD 5^th^ session F(48) = 3.7 p < 0.002**. Adult 2^nd^ session F(60) = 3.1, p < 0.001; 3^rd^ session F(60) = 3.6, p < 0.001; 4^th^ session F(60) = 4.0, p < 0.001; 5^th^ session F(60) = 3.6, p < 0.0001). **Excluding GDTs > 10 ms made this analysis uninterpretable for the MD group, as only three animals remained after exclusion.** There is still considerable within-animal variability across sessions, especially for the younger groups, suggesting a need for more experience to achieve consistent performance at younger ages. At a group level, this indicates a developmental trajectory for learning with non-attentional gap-PPI exposure. We confirmed that this improvement reflects learning rather than maturation of the auditory system by comparing age-matched animals with and without gap-PPI experience. The last two sessions of the MD group overlapped with the first two sessions of the LD group. Comparing performance from those sessions, the experienced MD animals had significantly better GDTs than the inexperienced LD animals (Figure 4A, Gap-PPI MD vs Gap-PPI LD: Kruskal-Wallis, best GDT: χ^2^(1,22) = 6.61, p < 0.02; average GDT: χ^2^(1,22) = 4.5, p < 0.035). Thus the improvement over testing sessions is due to gap-PPI experience rather than an effect of normal maturation.

***Effects of gap exposure alone on detection***

Improvement over sessions indicates learning based on exposure to gap-PPI testing, but does not distinguish what aspect of testing induces learning. We tested two additional groups of animals to determine whether the ASR testing method or simply exposure to gaps in background noise (unpaired with the startle stimulus) was sufficient to induce learning. To examine learning across sessions, we chose animals age-matched to the MD group, which required several sessions of experience to achieve consistently improved performance. Startle-Only MD animals were exposed to ASR testing in background noise without gaps for 3 sessions, then gap-PPI tested for the final 2 sessions. Thresholds for Startle-Only MD animals were no better than age-matched LD animals, who were equivalently naïve in terms of gap exposure (best GDT: χ^2^(1,26) = 0.11, p = 0.74; average GDT: χ^2^(1,26) = 0.11, p = 0.74). This indicates that experiencing ASR testing alone did not improve GDTs. Gap-Only MD animals were exposed to background noise *with* gaps but *without* ASR testing for 3 sessions, then gap-PPI tested for the final 2 sessions. **Based on both best and average of the two sessions**, these Gap-Only MD animals were significantly better than both the age-matched Startle-Only and Gap-PPI LD groups (Kruskal-Wallis: best GDTs: Gap-Only MD vs Startle-Only MD χ^2^(1,27) = 8.4, p < 0.004; Gap-Only MD vs Gap-PPI LD χ^2^(1,25) = 7.9, p < 0.005. **Average GDTs: Gap-Only MD vs Startle-Only MD χ^2^(1,27) = 6.7, p < 0.01; Gap-Only MD vs Gap-PPI LD χ^2^(1,25) = 4.2, p < 0.04**). Thus exposure only to gaps without PPI testing improved GDTs. Moreover, Gap-Only animals did not differ from the Gap-PPI MD group, showing that exposure only to gaps produced GDTs equivalent to gap-PPI testing (Kruskal-Wallis: best GDTs: Gap-Only MD vs Gap-PPI MD χ^2^(1,23) = 0.18, p = 0.67; average GDTs: Gap-Only MD vs Gap-PPI MD χ^2^(1,23) = 0.08, p = 0.78). This effect is visible as well in a less processed representation of the data, across the range of gap durations we tested (Figure 4B): the magnitude of the startle reduction by the gap was equivalent between Gap-PPI and Gap-Only MD animals at all gap durations. In contrast, the control Startle-Only MD animals had less of a startle reduction than either of these groups at most gap durations. Thus exposure to gaps in background noise unpaired with startles is sufficient to induce gap detection learning, and is as effective as gap-PPI testing.

***Effects of early exposure on adult detection***

Chronic exposure to auditory stimuli can have long-lasting perceptual effects during development but not in adulthood (Schreiner and Polley, 2014). These are typically detrimental to perception. Both our gap-PPI and gap-only results indicate that in short bouts, non-attentive exposure causes improvement, raising the question of long term effects. We tested whether gap-PPI testing has differential long-lasting effects based on the developmental period of exposure, with the idea that early exposure could improve later adult gap detection more than adult exposure (Sarro and Sanes, 2011). ED, MD, LD and Adult groups were re-tested for two sessions, 60 days after the initial 5 sessions (see timeline in Figure 1B). Figure 5A compares each animal’s initial GDT (the best of the first two sessions) with their final GDT (the best of the two +60-day sessions). This depicts adult gap detection (+60-day testing) after exposures at different developmental timepoints. The vast majority of animals improved (points below the diagonal) in the MD and LD age groups (Kruskal-Wallis, median improvement: MD: 3.5ms, χ^2^(1,20) = 5.1, p < 0.02; LD: 1.2ms, χ^2^(1,24) = 3.7, p = 0.05). **The adults did not significantly improve based on best GDTs, as they were already at ceiling (Kruskal-Wallis, median improvement: Adult: 0.4ms, χ^2^(1,30) = 0.5, p = 0.5)**. The final best GDTs of these three groups did not differ from one another, likely because all groups improved to ceiling performance (Figure 5B), obscuring any potential benefit of the age of initial exposure. MD, LD and adult groups all improved when including the added variability of averaged GDTs (Kruskal-Wallis, median improvement: MD: 6.7ms , χ^2^(1,20) = 8.8, p < 0.003; LD: 4.2ms, χ^2^(1,24) = 8.5, p < 0.003; Adult: 3.9ms, χ^2^(1,30) = 9.8, p < 0.002).

In contrast to the other three groups, 20% of the ED animals worsened in adult retesting (Figure 5A, points above the diagonal), and the median ED GDTs during adult retesting were significantly higher than those of the Adult group (Figure 5B; ED vs Adult: best GDT: χ^2^(1,70) = 17.4, p < 0.0001; average GDT: χ^2^(1,70) = 24.4, p < 0.0001). One possibility is that early exposure to the background noise with gaps may have caused a later gap detection deficit. To test this possibility, we added a control group (see timeline in Figure 1B). ED Startle-Only animals were exposed to 5 sessions of startle-only exposure during continuous background noise with no gaps, then gap-PPI tested at +60-days. At testing, their GDTs were equivalent to the ED group (ED vs ED Startle-Only, best GDT: χ^2^(1,68) = 0.03, p = 0.87; average GDT: χ^2^(1,68) = 0.81, p = 0.37), and significantly higher than those of Adult-exposed animals (Figure 5B; ED Startle-Only vs Adult best GDT: χ^2^(1, 30) = 8.6, p < 0.004; average GDT: χ^2^(1, 30) = 11.7, p < 0.001). Gap exposure thus did not cause worsening, as the Startle-Only animals worsened although they were not exposed to gaps.

Another possible explanation is that the startle exposure during testing may have induced hearing damage in the ED animals, as they were exposed to 110dB SPL startle sounds as early as P12. To test this possibility, we added another control group (see timeline in Figure 1B). ED Silence-Only animals were simply placed in the startle enclosure without exposure to any auditory stimuli for 5 sessions, then tested at +60-days. At testing, their GDTs were equivalent to the ED group (ED vs ED Silence-Only best GDT: χ^2^(1,69) = 0.4, p = 0.81; average GDT: χ^2^(1,69) = 3.8, p = 0.07), and significantly higher than those of Adult-exposed animals (Figure 5B; ED Silence-Only vs Adult best GDT: χ^2^(1,31) = 8.6, p < 0.004; average GDT: χ^2^(1,31) = 10.0, p < 0.002). These Silence-Only animals worsened although they were exposed to neither startle sounds nor gaps during development (their thresholds were only tested in adulthood). Thus, even if the other groups had hearing loss, this control indicates that worsening occurred in the absence of hearing loss. It is noteworthy that in all ED groups, many individuals performed as well as Adult exposed animals; our manipulations impaired later GDTs only in a subset of individuals.

An alternative explanation for the worsening involves non-auditory elements of testing. Maternal separation at a very young age has been demonstrated to induce long-lasting behavioral deficits and is a well-established stress model (Francis et al., 2002; Nishi et al., 2014; Schäble et al., 2007). One known effect of early acute stress is a higher response magnitude to startle-only stimuli (Bakshi et al., 1998). To test whether the ED animals displayed increased startle-only magnitudes, we compared the response magnitudes to startle-only stimuli across age-matched groups with different early experience (Figure 5C and timeline in Figure 1B). (The age-match is necessary because animal mass affects startle magnitude, although it does not affect GDT as seen in Fig 2B inset.) All ED groups experienced early testing and the possible stress it could induce. ED Silence-Only animals tested at +60-days, who were naïve to startle stimuli unlike the other two ED groups, had significantly higher startle response magnitudes than those two groups (ED Silence-Only vs ED χ^2^(1,70) = 37.3, p < 0.0001; ED Silence-Only vs ED Startle-Only χ^2^(1,30) = 27.8, p < 0.0001). This reflects a sensitized but transient response to startle, enhanced upon first experiencing startle sounds and back to normal levels after multiple startle sessions. Importantly, this large response magnitude was not due to naïvete to startles: the ED Silence-Only group had significantly higher startle response magnitudes than the age- and experience-matched Adult group who were equivalently naïve to startles (χ^2^(1,29) = 31.2, p < 0.0001).
